# Changes in circRNA expression profiles related to the antagonistic effects of *Escherichia coli* F17 in lamb spleens

**DOI:** 10.1038/s41598-018-31719-5

**Published:** 2018-09-28

**Authors:** Chengyan Jin, Jianjun Bao, Yue Wang, Weihao Chen, Shuangxia Zou, Tianyi Wu, Lihong Wang, Xiaoyang Lv, Wen Gao, Buzhong Wang, Guoqiang Zhu, Guojun Dai, Dongfang Shi, Wei Sun

**Affiliations:** 1 Key Laboratory for Animal Genetics, Breeding, Reproduction and Molecular Design of Jiangsu Province, College of Animal Science and Technology, Yangzhou University, Yangzhou, 225009 Jiangsu P. R. China; 2 Jiangsu Xilaiyuan Ecological Agriculture Co., Ltd. Taizhou, Taizhou, 225300 Jiangsu P. R. China; 3 College of Veterinary Medicine, Yangzhou University, Yangzhou, 225009 Jiangsu P. R. China; 4 College of Veterinary Medicine, Northeast Agricultural University, Harbin, 150030 P. R. China

## Abstract

Sheep colibacillosis is one of the most common bacterial diseases in large-scale sheep farms. In this study, we orally administered *Escherichia coli* F17 (*E. coli* F17) to lambs to obtain antagonistic and sensitive individuals. We used RNA-seq to screen for differential circRNAs in the spleens of both antagonist and sensitive individuals to explore the effect of circRNA on anti-diarrhoea in sheep. The results showed that 60 differentially expressed (DE) circRNAs were screened by RNA-seq in the spleen of antagonistic and sensitive lambs, among which 31 were up-regulated and 29 were down-regulated; q-PCR was used to validate the relative expression levels of six randomly selected circRNAs in antagonist and susceptible lambs and found to be consistent with the results of RNA-seq. Using Miranda analysis of circRNA-miRNA-mRNA interactions, we found a certain target relationship between 6 circRNAs, 5 miRNAs and 9 mRNAs. The relative expression levels of mRNA in antagonistic and sensitive lambs were verified by q-PCR and were consistent with the results of RNA-seq. This study explored the expression profile of circRNA in the spleen of an antagonistic and susceptible lamb with diarrhoea and found that differentially expressed circRNAs were helpful for determining how the lambs resist the pathogenesis of diarrhoea and provided a scientific basis for lambs to resist diarrhoea.

## Introduction

Sheep colibacillosis is one of the most common bacterial diseases in large-scale sheep farms. The traditional method of controlling the bacterial disease is by antibiotic therapy, although this approach also has several disadvantages. The use of RNA-seq to screen circRNAs that antagonize sheep colibacillosis is the basis for analysing the molecular mechanism of disease resistance in sheep and thus for discovering candidate genes associated with disease resistance traits. Circular RNA (circRNA) is a type of special non-coding RNA (ncRNA), which is a new research hotspot, that is in a RNA family along with microRNA (miRNA) and long non-coding RNA (lncRNA)^1^. Using electron microscopy, Sanger *et al*.^2^ first found the closed circular single-stranded RNA molecules formed by covalent bonds in plant-infected viruses (viroids), which are highly thermostable. In 1990, researchers found that 20S RNA did not have free 5′ and 3′ ends in Saccharomyces cerevisiae, and it was determined by electron microscopy to be a circular RNA molecule^3,4^. Subsequently, circRNAs were found in the hepatitis D virus^5^, and circRNAs transcribed from the sex-determining region Y (Sry) were also found in the testes of mice^6^. It was also confirmed that circRNAs are present in human cells^7^.

Although circRNAs are widely found in a variety of cells over a long period of time, the study of circRNA has been slow over the past few decades, and the mechanism of gene expression and regulation is not fully understood^8^. For a long time, circRNAs were identified as by-products of alternative splicing during pre-mRNA processing. However, only a few circRNAs were found to be exons produced during alternative splicing^9^. Recent studies^10^ have found that circRNA is not a by-product of mRNA maturation and, like mRNA, is an important product of pre-mRNA processing; circRNA processing machinery competes with mRNA. At the same time, the classical clipping signal and clipping mechanism are also necessary for back-shear^11^. At present, studies on sheep disease resistance mainly focus on the prevention and treatment of disease^12,13^, but the important molecular mechanisms of disease resistance are seldom reported. In this study, we first screened for circRNAs that were differentially expressed in individuals that were antagonistic and sensitive to *E. coli* F17 fimbriae by RNA-seq. Miranda was used to analyse the circRNA-miRNA-mRNA interaction to find miRNA target genes and then verified by q-PCR. At the circRNA level, this study deepens our understanding of antagonizing *E. coli* F17 fimbriae in sheep and at the same time is expected to identify genes that can antagonize *E. coli* F17 fimbriae and solve key problems for Chinese local sheep breeding against *E. coli* disease. This study can be used as a foundation and can provide a theoretical basis for formulating a breeding strategy against *E. coli* in the future.

## Results

### Identification of transcripts in sheep spleens

**Figure 1:**
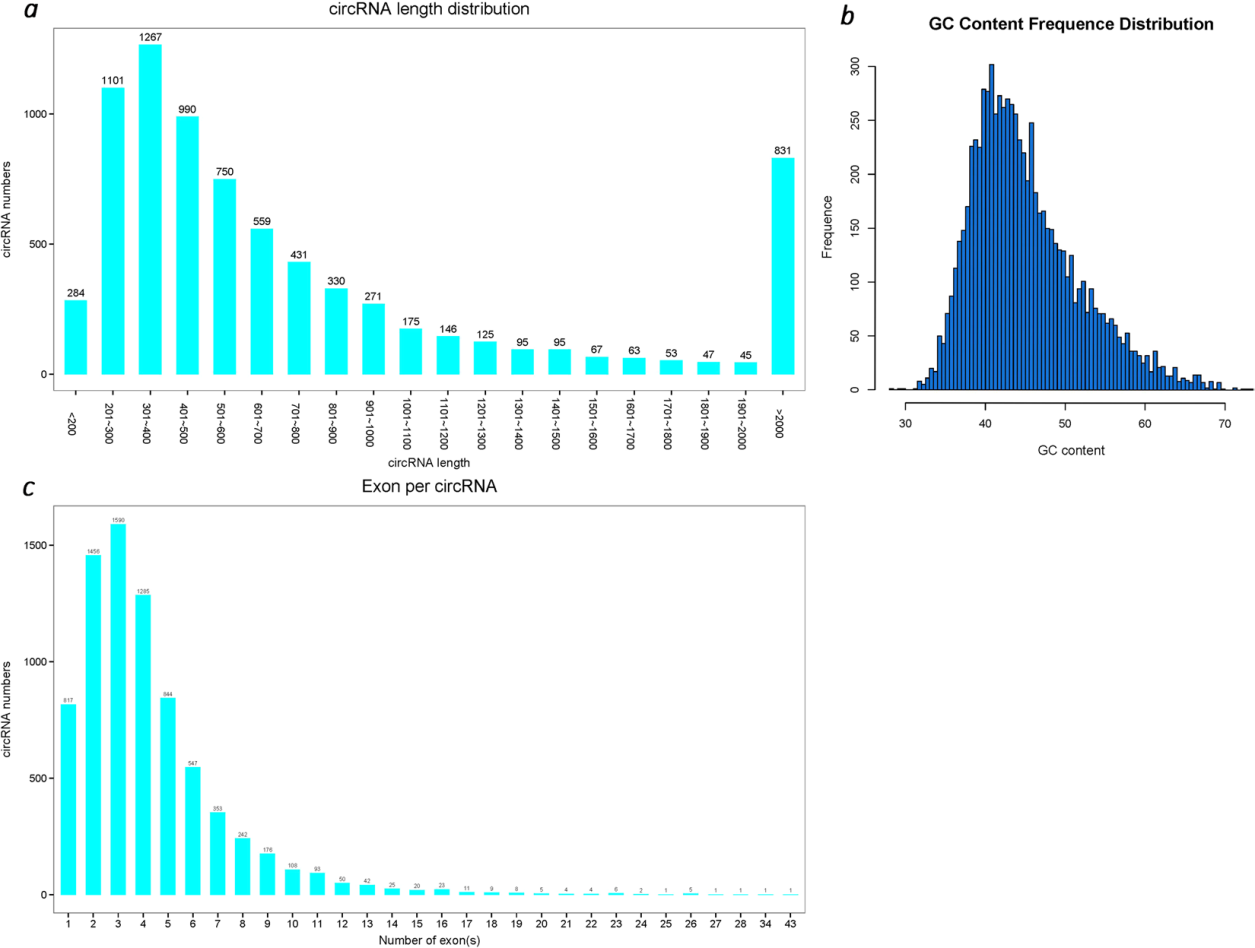
Summary of the length, GC content, and number of the exons of the predicted circRNAs. (**a**) Shows the length of circRNAs, which primarily ranges from 200 bp to 900 bp, with an average length of 1943 bp. (**b**) Shows a GC content of approximately 43.5%. (**c**) Shows the number of exons of circRNA, which is mainly 2–4.

**Figure 2:**
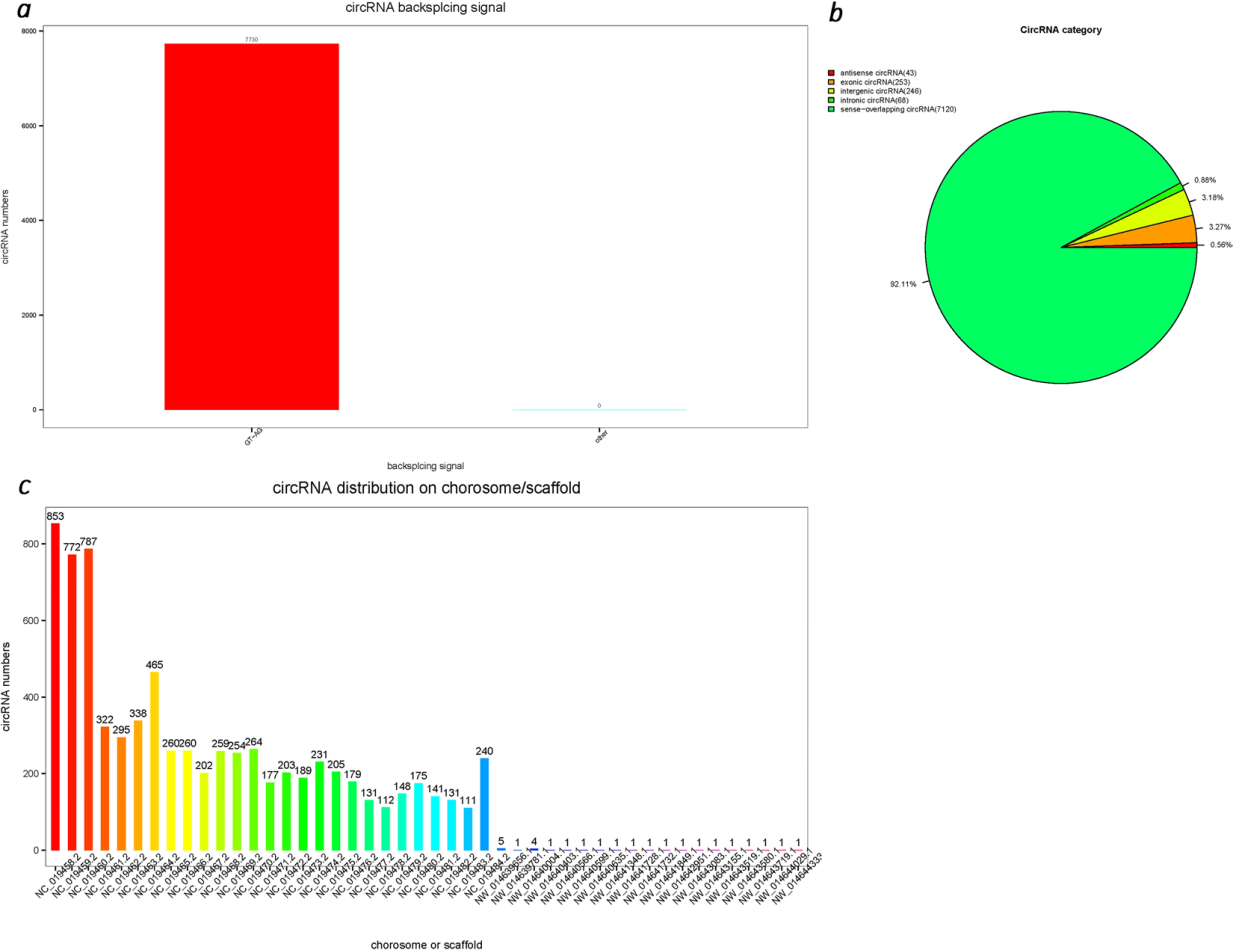
circRNA cleavage site signal statistics, circRNA gene structure distribution and circRNA number distribution in each chromosome or the scaffold. (**a**) Shows the statistics of the variable shear signal (GT-AG) of the reverse cleavage site in the circRNA sequence. (**b**) Shows the statistics for the circRNA types, overlapping circRNA accounts for 92.11%, exonic circRNA accounts for 3.27%, intergenic circRNA accounts for 3.18%, intronic circRNA accounts for 0.88% and antisense circRNA accounts for 0.56%. (**c**) shows the number of circRNAs predicted on each chromosome or scaffold.

After mapping the reference sequence, we identified 7,730 known circRNAs. The length of circRNAs primarily ranges from 200 bp to 900 bp, with an average length of 1943 bp and a GC content of approximately 43.5%. The number of exons of circRNA is mainly 2–4 (Fig. 1). The statistics of the variable shear signal (GT-AG) of the reverse cleavage site in the circRNA sequence were calculated and graphs were drawn (Fig. 2a). The statistics for the circRNA types are shown in Fig. 2b: overlapping circRNA accounts for 92.11%, exonic circRNA accounts for 3.27%, intergenic circRNA accounts for 3.18%, intronic circRNA accounts for 0.88% and antisense circRNA accounts for 0.56%. circRNAs were compared to the genomic elements to explore the distribution of circRNAs in the genome, to count the number of circRNAs predicted on each chromosome or scaffold, and to plot the results (Fig. 2c). It was found that circRNAs were primarily distributed on three chromosomes: NC_019458.2 (853), NC_019459.2 (772), NC_019460.2 (787).

### Analysis and validation of DE transcripts

**Figure 3:**
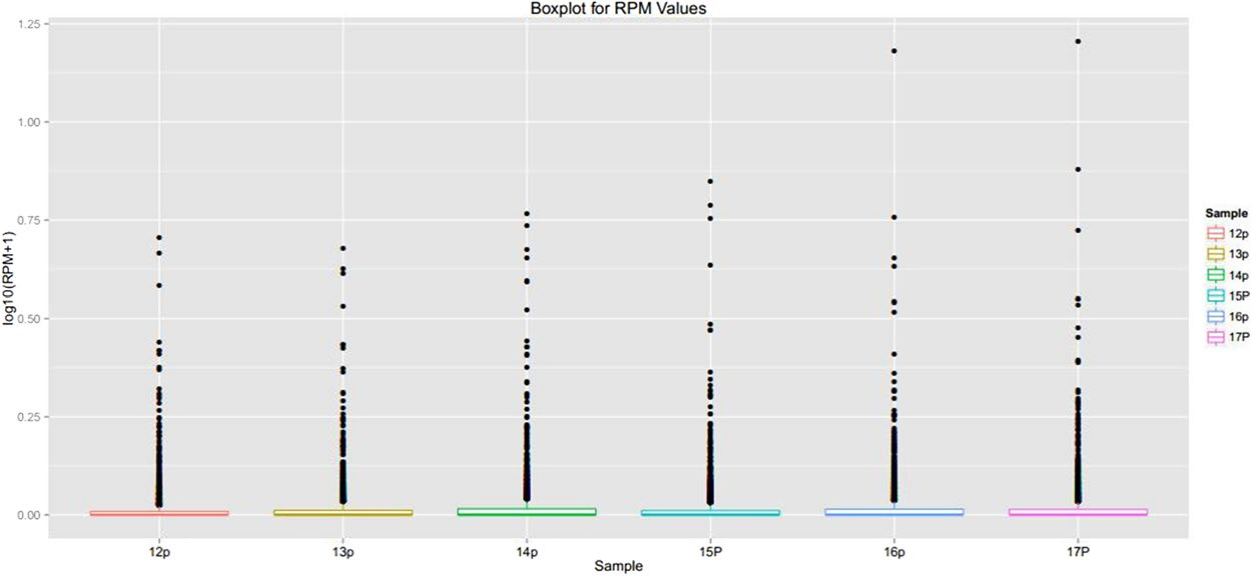
Expression patterns of circRNA transcripts, The Box-whisker Plot consists of five statistics: the minimum, the first quartile (25%), the median (50%), the third quartile (75%), and the maximum.

**Figure 4:**
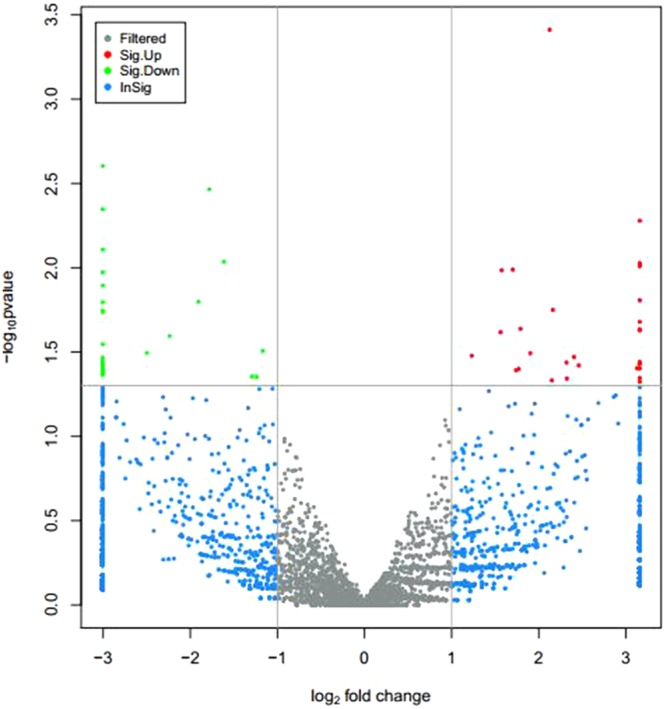
Differentially expressed circRNAs in antagonistic and sensitive lambs Note: Gray represents circRNAs that have no significant differences; Red represents significantly upregulated circRNAs; Green represents significantly downregulated circRNAs; Blue indicates that the difference multiple is more than 2 times, but the circRNA is not significant in the difference significance test. The horizontal axis is the display of log2 FoldChange, and the vertical axis is the display of log10 Pvalue.

**Figure 5:**
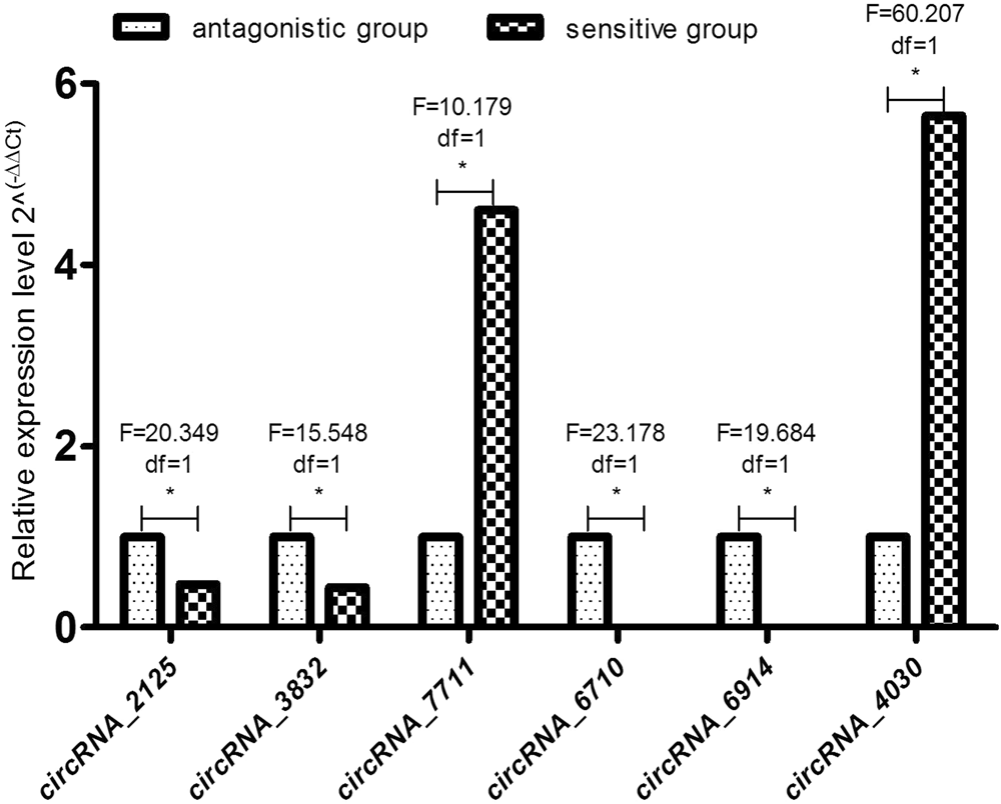
Relative expression levels of DE circRNAs between antagonistic and sensitive lambs Note: “**” means highly significant correlation; “*” means significant correlation; “ns” or “no SuperiorScript” means no significant correlation. The same as below.

We used the RPM value to estimate the expression level of circRNA transcripts, and the expression level of circRNA transcripts was low (Fig. 3). We screened 31 up-regulated and 29 down-regulated DE circRNAs (Fig. 4). Differentially expressed circRNAs can be found on Table 1. To further validate the reliability of RNA-seq, 6 DE circRNAs were randomly selected, and their relative expression levels in antagonistic and sensitive lambs were confirmed by q-PCR and found to coincide with our RNA-seq results (Fig. 5), thus indicating that the RNA-seq data are reliable. Our analyses also show that high-throughput sequencing has the advantage of detecting genes with low expression levels.

**Table 1:** Differentially expressed circRNA.

id	baseMean_control_Group_S	baseMean_case_Group_R	foldChange	pval	up_down
circRNA_0093	0	3.328674397	Inf	0.036236093	Up
circRNA_1685	0	4.142718429	Inf	0.009371524	Up
circRNA_1940	0	3.60566823	Inf	0.023219032	Up
circRNA_2187	0	4.588882563	Inf	0.005265342	Up
circRNA_2369	0	4.081784422	Inf	0.00975272	Up
circRNA_3123	0	3.186563169	Inf	0.039499236	Up
circRNA_3214	0	4.081784422	Inf	0.00975272	Up
circRNA_3620	0	3.794668982	Inf	0.015587663	Up
circRNA_4030	0	5.299025941	Inf	0.020922639	Up
circRNA_6527	0	3.588730763	Inf	0.023459223	Up
circRNA_7034	0	3.588730763	Inf	0.023459223	Up
circRNA_7304	0	3.284677857	Inf	0.037195563	Up
circRNA_7332	0	2.91246232	Inf	0.047349658	Up
circRNA_7711	0	5.177157926	Inf	0.036925058	Up
circRNA_6529	0.547672354	4.890042486	8.928773656	0.045003571	Up
circRNA_3185	0.547672354	4.791927799	8.749625143	0.039389965	Up
circRNA_2522	1.149706435	6.326236815	5.502480129	0.037974962	Up
circRNA_0523	1.042237174	5.515085766	5.291584204	0.0338266	Up
circRNA_2875	1.149706435	5.748083059	4.999609363	0.045365948	Up
circRNA_4546	1.042237174	5.196988376	4.986377868	0.036464063	Up
circRNA_4243	1.916853239	8.569659314	4.470691412	0.017794489	Up
circRNA_3264	1.242497137	5.51797875	4.44103941	0.046575522	Up
circRNA_5016	5.184927069	22.61330084	4.361353696	0.000387929	Up
circRNA_7258	2.025576694	7.572400497	3.73839239	0.032119531	Up
circRNA_3864	2.339096038	8.089620246	3.458438694	0.023022668	Up
circRNA_7704	5.47672354	18.67362764	3.409634886	0.039858093	Up
circRNA_2835	2.246305337	7.4984519	3.338126735	0.040473314	Up
circRNA_6899	4.436994753	14.43279453	3.252831101	0.010250326	Up
circRNA_1130	4.556966317	13.56649544	2.977089251	0.010346567	Up
circRNA_6542	3.661881572	10.80296021	2.950111847	0.024097087	Up
circRNA_4545	7.431232406	17.44626452	2.347694644	0.033248561	Up
circRNA_7016	25.14782676	11.19500619	0.445167939	0.03112797	Down
circRNA_2198	13.64497228	5.792079599	0.424484527	0.044388965	Down
circRNA_6187	11.06718731	4.514933966	0.407956768	0.044162581	Down
circRNA_4841	19.28728939	6.298147848	0.32654396	0.009214312	Down
circRNA_7412	16.31897841	4.747931258	0.290945373	0.003424958	Down
circRNA_7434	10.10649272	2.696402494	0.266799034	0.015887585	Down
circRNA_0771	6.822486141	1.446315935	0.211992506	0.025384118	Down
circRNA_0299	5.900220532	1.044148341	0.17696768	0.031991668	Down
circRNA_1040	4.885164222	0.608105813	0.124480117	0.04313322	Down
circRNA_0309	23.67770538	0	0	0.002492483	Down
circRNA_1291	3.916503262	0	0	0.018259526	Down
circRNA_1712	3.388045395	0	0	0.038693764	Down
circRNA_2125	6.073299631	0	0	0.000933537	Down
circRNA_3252	4.001327587	0	0	0.034258523	Down
circRNA_3323	3.341650045	0	0	0.039900795	Down
circRNA_3594	4.840023064	0	0	0.02843827	Down
circRNA_3832	385.287501	0	0	0.034358494	Down
circRNA_3974	4.411068082	0	0	0.010635084	Down
circRNA_4396	3.442407123	0	0	0.03735108	Down
circRNA_5477	3.388045395	0	0	0.038693764	Down
circRNA_6144	4.143944089	0	0	0.012739266	Down
circRNA_6165	5.051531137	0	0	0.004500814	Down
circRNA_6403	4.110051041	0	0	0.016030151	Down
circRNA_6561	3.295254694	0	0	0.041167962	Down
circRNA_6577	3.380079018	0	0	0.042556277	Down
circRNA_6710	3.488802473	0	0	0.036261222	Down
circRNA_6914	4.556966317	0	0	0.007791152	Down
circRNA_7559	3.240892967	0	0	0.04273448	Down
circRNA_7725	7.03993305	0	0	0.017985334	Down

### GO and KEGG pathway enrichment analyses of DE lncRNAs

**Figure 6:**
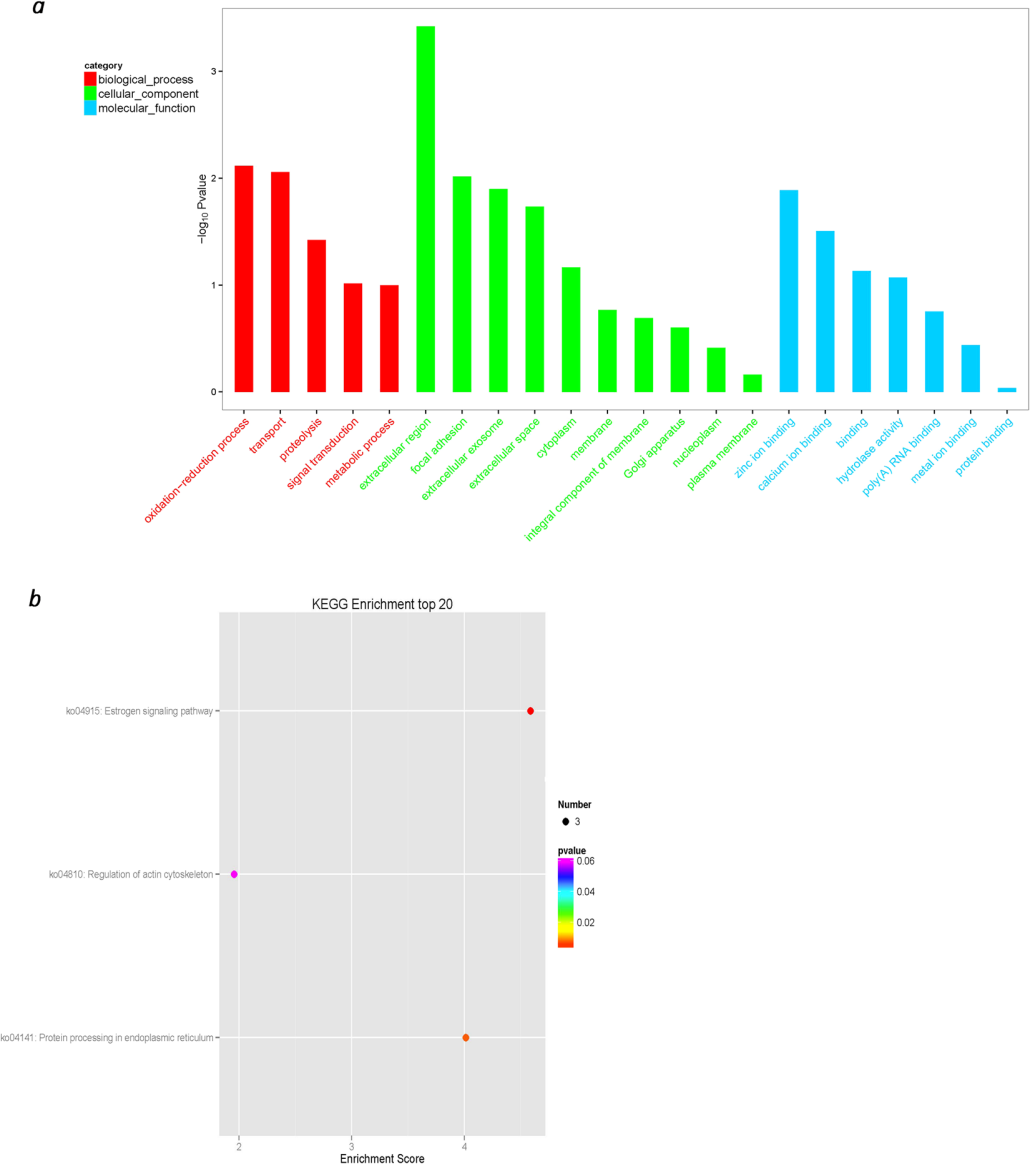
Gene Ontology and KEGG pathway enrichment analyses of DE circRNAs.

A comparison of the DE circRNA and GO databases showed that a total of 60 circRNAs were annotated and classified into 297 functional subclasses. The results showed the oxidation-reduction process (GO: 0055114), transport (GO: 0006810), extracellular region (GO: 0005576), focal adhesion (GO: 0005925), extracellular exosome 0005615), zinc ion binding (GO: 0008270) and seven more subclasses of circRNA functions, while the remaining functional subclass circRNA was less distributed (Fig. 6a). A comparison of the DE circRNA and KEGG PATHWAY databases showed that a total of 60 circRNAs were annotated and grouped into 73 KEGG PATHWAYS. The results showed that there were more circRNAs in three KEGG pathways, including the estrogen signalling pathway (path: ko04915), protein processing in the endoplasmic reticulum (path: ko04141) and regulation of the actin cytoskeleton (path: ko04810). The remaining KEGG pathways have fewer circRNAs (Fig. 6b).

### Prediction of target relationship of circRNA-miRNA-mRNA

**Figure 7:**
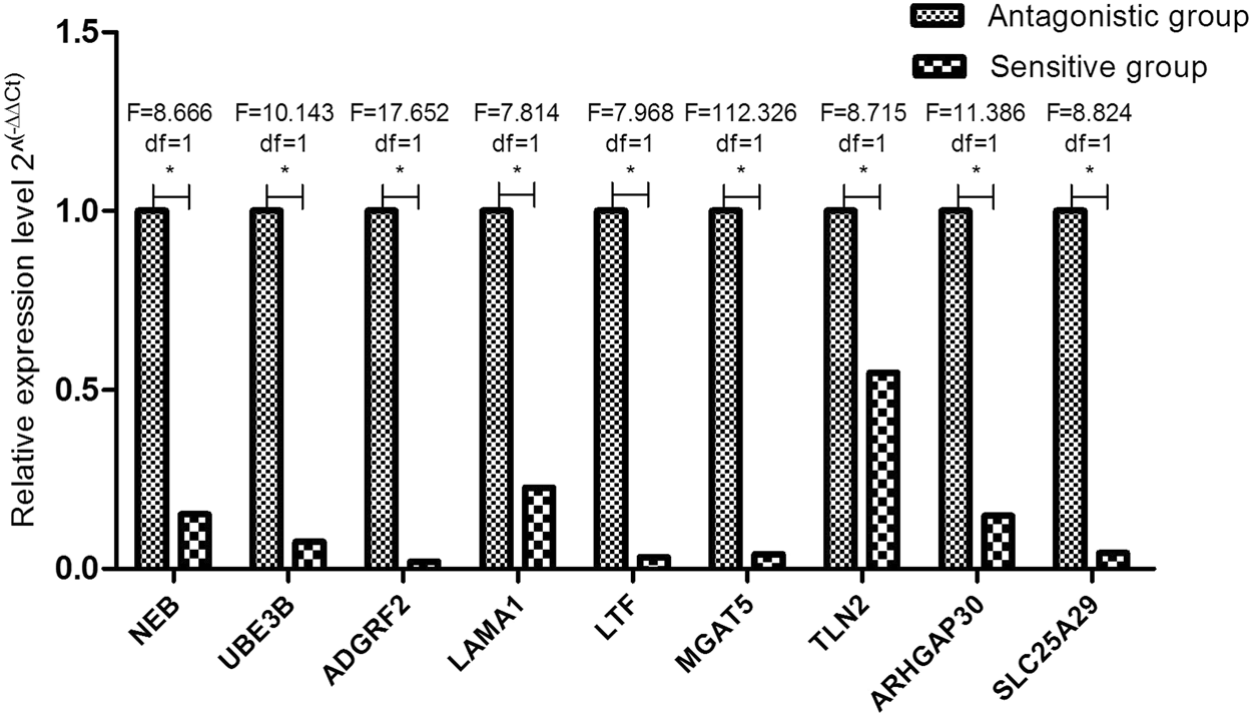
Relative expression level of the target genes of miRNAs between antagonistic and sensitive lambs.

We used Miranda to predict miRNA-bound circRNA and the target genes of the miRNA. The function of circRNAs was elucidated on the basis of the function of the target genes of miRNAs. The predicted results are shown in Table 2 below. To further validate the relative expression, 9 mRNAs were selected, and their relative expression levels in antagonistic and sensitive lambs were confirmed by q-PCR. Significant differences were found between the two groups (Fig. 7).

**Table 2:** Prediction of the target relationship of circRNA-miRNA-mRNA.

circRNA	Best gene of circRNA	pval	miRNA	Target gene of miRNA	Transcription ID of Target gene
circRNA_6577	LOC101111058 (Btnl 1)	0.000190825	oar-miR-381-5p		
circRNA_7725		0.003873598	oar-miR-1193-5p	NEB UBE3B	XM_012137591.2 XM_004017436.3
circRNA_0309	LOC101108092 (GSTM1)	0.004205007	oar-miR-370-3p	ADGRF2	XM_004018870.3
circRNA_2125	LOC101115614 (NRAMP2)	0.004205007	oar-miR-370-3p	LAMA1	XM_012103553.2
circRNA_3832	B2M	0.004205007	oar-miR-370-3p	LTF	NM_001024862.1
circRNA_6577	LOC101111058 (Btnl 1)	0.004205007	oar-miR-370-3p	MGAT5	XM_012139230.2
circRNA_7711		0.004205007	oar-miR-370-3p	TLN2	XM_012181407.2
circRNA_6577	LOC101111058 (Btnl 1)	0.006550584	oar-miR-3956-3p	ARHGAP30 SLC25A29	XM_012184619.2 XM_015102051.1
circRNA_6577	LOC101111058 (Btnl 1)	0.011765813	oar-miR-370-5p

## Discussion

Due to the limited efficiency of traditional molecular biology methods for the detection of circRNAs, circRNA has long been regarded as a product of the abnormal splicing of RNA^7^. In recent years, with the rapid development of bioinformatics and high-throughput sequencing technology, a large number of circRNAs have been identified in eukaryotes^6,14,15^, and they may play an important role in the regulation of gene expression^9,16,17^. It has been found that most circRNAs contain miRNA binding sites that act as efficient competitive endogenous RNAs and efficiently adsorb miRNAs to regulate the target genes of miRNA^18,19^, which have the following four biological functions: miRNA sponge effect^17,18^, protein translation template^20,21^, regulation of gene transcription^22,23^ and regulation of competition for linear RNA production^8,10^. However, investigations of lamb diarrhoea relative to circRNAs are limited. Hu sheep are a unique breed with high fecundity and a strong adaptability to warm-wet climates; they can be kept indoors all year round. This study provides the first overview of circRNAs in relation to diarrhoea in sheep, as well an investigation into their possible roles in disease resistance.

In the present study, we found that the expression level of circRNA was very low: 5 miRNAs binding to 6 circRNAs were identified by the Miranda software, and the adjacent genes of 4 circRNAs were identified as *Btnl 1*, *GSTM1*, *NRAMP2* and *B2M*.

*Btnl 1* is a key suppressor of T-cell activation and immune diseases^24^. The mechanism of action of *Btnl 1* is different from those of *Btnl 2* and *BtnlA1*, which directly inhibit T cell activation through anti-receptor binding^24–27^. The study found that the Btnl gene may be a new important local regulator of intestinal inflammation^28^. *GSTM1* encodes the glutathione-S-transferase (GST) M1 enzyme, which is involved in the detoxification of various carcinogens of lung cancer^29^ and plays a key role in protecting cells from oxidative stress^30^. *NRAMP2* is a metal transporter protein, and in the absence of manganese, *NRAMP2* is involved in the regeneration of Mn in the Golgi and promotes plant root growth^31^. *B2M* encodes the beta chain of the major histocompatibility complex (MHC) class I molecule and is up-regulated in inflammatory and tumour cells^32^.

We also used Miranda software to predict the 3 miRNA target genes and verified that they were significantly differentially expressed in antagonistic and sensitive groups, namely, *NEB*, *UBE3B*, *ADGRF2*, *LAMA1*, *LTF*, *MGAT5*, *TLN2* and *SLC25A29*.

*NEB* encodes nebulin, a large protein component of the cytoskeletal matrix that coexists with myofilaments in skeletal muscle. Mutations in the NEB gene are the most common causes of myotubes, accounting for approximately 50%^33^. *UBE3B* is a ubiquitin ligase (*UBE3*), and its unique combination of E2-binding enzymes provides high substrate specificity, which is required to target specific protein degradation^34^. *ADGRF2* is a member of the adhesion G protein-coupled receptor family and plays an important role in adhesion in the cell-cell and cell-matrix^35^. *LAMA1* mutations may be related to Poretti-Boltshauser syndrome, and studies have shown that *LAMA1* deficiency can lead to cytoskeletal changes^36^. *LTF* is a member of the transferrin family, whose protein products initiate host defence against a wide range of microbial infections and antigenic activity^37^. The protein encoded by *MGAT5* belongs to the glycosyltransferase family and is one of the most important enzymes involved in the regulation of the biosynthesis of glycoprotein oligosaccharides. Changes in oligosaccharides on cell surface glycoproteins cause significant changes in cell adhesion or migration behaviour; increased enzyme activity is associated with the development of invasive malignancies^38^. Talin is a large adapter protein that links the integrin family of adhesion molecules to F-actin; Talin 1 is required for integrin-mediated cell adhesion, and *TLN2*, like Talin 1, is considered to be unique. Transmembrane receptors bind to form new connections between the extracellular matrix and the actin cytoskeleton^39^. *SLC25A29* encodes a nuclear-encoded mitochondrial protein that is a member of the large family of solute transporter family 25 (*SLC25*) mitochondria. The primary physiological role of *SLC25A29* is to introduce basic amino acids into the mitochondria for mitochondrial protein synthesis and amino-acid degradation^40^.

GO is a bioinformatics tool that is widely used to study the functional relationship of genes. GO and KEGG pathway analyses of 60 DE circRNAs showed that the relevant circRNAs may potentially participate in the process of pili adhesion to intestinal mucosa. However, the role of these pathways in disease resistance remains largely unknown.

We found that a total of 60 circRNAs were significantly differentially expressed between antagonistic and sensitive groups, with 31 up-regulated and 29 down-regulated. In addition, we identified a total of 1,942 new circRNAs in both groups. To further verify the results of RNA-Seq, the expression levels of the six circRNAs were verified by q-PCR, and the results were consistent.

We studied the expression profiles of circRNAs in the spleens of antagonistic and sensitive sheep that developed diarrhoea to further understand their regulatory role in disease resistance in sheep. We found that circRNAs were differentially expressed in spleen tissues of antagonistic and sensitive lambs. Our research may help to determine how lambs resist the mechanism of diarrhoea. In addition, further studies of these circRNAs can provide a scientific basis for lambs to resist diarrhoea.

## Methods

### Ethics statement

The Institutional Animal Care and Use Committee (IACUC) of the government of Jiangsu Province (Permit Number 45) and Ministry of Agriculture of China (Permit Number 39) approved the animal study proposal. All experimental procedures were conducted in strict compliance with the recommendations of the Guide for the Care and Use of Laboratory Animals of Jiangsu Province and of the Animal Care and Use Committee of the Chinese Ministry of Agriculture. All efforts were made to minimize animal suffering.

### Experimental design and sample collection

Experimental sheep were purchased from Jiangsu Xilaiyuan Ecological Agriculture Co., Ltd. in December 2016. A total of 18 three-day-old lambs showing normal growth and roughly similar weight were randomly selected, and all sheep were raised with segregation. To ensure their dietary requirements were met, all sheep were fed with 10% lamb milk powder prior to the experiment. Five-day-old lambs were fed 12.5% lamb milk powder and *E. coli* F17 bacteria liquid [4.6 × 10^8^ colony-forming units (CFUs)·mL^−1^]^41^ and had *ad libitum* access to drinking water. The stool features^42^ of the experimental lambs were recorded daily. Lambs that exhibited diarrhoea for two days were classified as antagonistic and sensitive and then euthanized. The intestinal tissues were collected in 4% paraformaldehyde. The liver, spleen, duodenum, jejunum, and ileum of each lamb were collected and immediately frozen in liquid nitrogen until RNA extraction.

### Library construction and sequencing

RNA was extracted from the spleen of three individuals per group. A NanoDrop 2000 Ultra Microscope and an Agilent 2100 Bioanalyser (Shanghai, China) were utilized for quality control of the extracted total RNAs (Annex 1). Ribosomal RNA was removed using a Ribo-Zero (TM) kit (Epicenter, Madison, WI, USA). Short fragments (approximately 200 bp in length) were obtained and used as templates for first-stand cDNA synthesis. Second-strand cDNA synthesis was performed using a buffer, dNTPs, RNase H, and DNA polymerase I. After PCR amplification and purification using the Qubit® dsDNA HS Assay Kit, the cDNA library was constructed using an NEBNext® Ultra™ RNA Library Preparation Kit. The cDNA library was sequenced on the Illumina HiSeq2500 platform by Shanghai OE Biomedical Technology Co. (sequencing read length: 150 bp).

### Identification of circRNAs

The circBase^43^ database contains only circRNA sequences of human, mouse, nematode, lagomorph, and coelacanth. Since sheep are not included, we used CIRI^44^ de novo prediction of circRNA. According to the position of circRNA on the genome, circRNAs can be classified into the following five categories: exonic circRNA, intronic circRNA, antisense circRNA, sense overlapping circRNA, and intergenic circRNA.

### Different expression analysis

After obtaining differentially expressed circRNAs, we performed Gene Ontology (GO) and KEGG pathway significance analyses of the source genes. DESeq^45^ is suitable for experiments with biological duplication. Differential expression analysis can be performed between sample groups to obtain the circRNA list for the difference between the two biological conditions. For experiments without biological duplication, edgeR^46^ differential expression analysis was used to obtain a list of circRNAs that were differentially expressed between the two samples.

### GO and KEGG pathway analyses

After screening for differentially expressed transcripts, functional annotation was performed using GO enrichment analysis. Enrichment analysis involved counting the number of transcripts in each GO term, followed by Fisher’s exact test to assess statistical significance (p < 0.05). KEGG^47^ is the main public database used in pathway analysis, which was followed by Fisher’s exact test to assess statistical significance (p < 0.05).

### circRNA-miRNA-mRNA interaction studies

As a miRNA target molecule, circRNAs are regulated by miRNAs. Because circRNAs contain multiple miRNA binding sites, analysis of circRNA-miRNA interactions can help elucidate the function and mechanism of circRNA acting as a sponge. For animals, we used Miranda^48,49^ to predict circRNAs that bind to miRNAs and the target genes of miRNA and elucidated the function of this portion of circRNAs based on the functional annotations of miRNA target genes.

### Verification of the expression level of DE circRNAs

To verify whether the screened DE circRNAs play a role in the process of antagonism, q-PCR was used to detect the expression levels of DE lncRNAs and target genes of DE miRNAs in lamb spleens between the antagonistic and sensitive groups. The relative expression of each RNA was normalized to that of GAPDH using the 2^−ΔΔCt^ method^50^, and the primers used in the amplification of the lncRNAs are shown in Table 3.

**Table 3:** Primers of *GAPDH*, DE circRNAs, and the target genes of DE miRNAs.

	Gene symbol	Primer sequence	Length of product/bp
Primers of DE circRNA	circRNA_2125	F:ATTGAATCACTTCTCTGTTGC	129
		R:TAGGTGCTCAAAATAGGAC	
	circRNA_3832	F:AGCCTCTCATCTGTACAC	134
		R:CAGTAACTGCCTAGAGCA	
	circRNA_7711	F:ACAAAGATTCCATTGACAG	101
		R:ACCAAGAGGCTAGCAAGAC	
	circRNA_6710	F:CAGATTACAGCTATGGCGA	124
		R:CCCTCATGATCTCATAGG	
	circRNA_6914	F:TTGGCTGTTACTATCATGAG	124
		R:CTGAACTCTTAACTTGCA	
	circRNA_4030	F:TGATGCAGATATTAAACCTC	133
		R:CCAATCTCGGATAACTTCAC	
Primers of the target genes of miRNAs	NEB	F:ATTACAGCTATCCACCCGAC	149
		R:TGCCTTTTCCATTTCTAAG	
	UBE3B	F:TAAGATTGCCAGGAAACTGC	133
		R:AGCCAGGGACACGTACCAC	
	ADGRF2	F:GGCGTTTACCTCTTTCTCG	103
		R:CAAGCTGCAAATAGAAAC	
	LAMA1	F:AAATGATCGAAAAGGCTAC	127
		R:AACCGCCTTTTCCGTAGGAC	
	LTF	F:GAAAAGCGTATCCCAACCTG	103
		R:TTGAAGGCACCAGAATAAC	
	MGAT5	F:CATCATCCACACCTACACG	111
		R:AACTGCAAGTCTCGTCCGC	
	TLN2	F:ACGACGGTGGTTAAATAC	125
		R:AGTTGCCCATAGTCACTGGTC	
	ARHGAP30	F:TCTTCAACCTGGGTCGCTC	159
		R:GCAGCCCCTCTGGTTCATC	
	SLC25A29	F:GCGTCCTGGCTCTCCACCT	125
		R:CCCTGCCTCCCCGCGCTC	
Primers of GAPDH	GAPDH-F	F:GTTCCACGGCACAGTCAAGG	127
		R:ACTCAGCACCAGCATCACCC	

### Statistical analysis

All data were analysed by SPSS (version 22.0), and the relative expression levels of various transcripts were analysed by one-way ANOVA. Statistical significance was determined when p < 0.05. Each group contained three samples, and each experiment was repeated three times.

## Data Availability

We guarantee that our data is valid.
